# Exposure to valproic acid is associated with less pulmonary infiltrates and improvements in diverse clinical outcomes and laboratory parameters in patients hospitalized with COVID-19

**DOI:** 10.1371/journal.pone.0262777

**Published:** 2022-01-27

**Authors:** Julio Collazos, Pere Domingo, Nerio Fernández-Araujo, Elia Asensi-Díaz, Helem Vilchez-Rueda, Antonio Lalueza, Emilia Roy-Vallejo, Rosa Blanes, Manuel Raya-Cruz, Jaime Sanz-Cánovas, Arturo Artero, José-Manuel Ramos-Rincón, Carlos Dueñas-Gutiérrez, José Luis Lamas-Ferreiro, Víctor Asensi, the Valproic Acid in COVID-19 Study Group

**Affiliations:** 1 Infectious Diseases Section, Hospital de Galdakao-Usansolo, Galdakao, Vizcaya, Spain; 2 Infectious Diseases Department, Hospital de la Santa Creu i Sant Pau, Barcelona, Spain; 3 Intensive Care Unit, Hospital General Universitario Gregorio Marañón, Madrid, Spain; 4 Internal Medicine Service, Hospital Universitario Fundación Jiménez Díaz, Madrid, Spain; 5 Infectious Diseases Section, Hospital Universitari Son Espases, Palma de Mallorca, Spain; 6 Internal Medicine Service, Hospital 12 de Octubre, Madrid, Spain; 7 Infectious Diseases Section, Hospital Universitario La Princesa, Madrid, Spain; 8 Internal Medicine Service, Hospital Universitario y Politécnico La Fe, Valencia, Spain; 9 Internal Medicine Service, Hospital Son Llàtzer, Palma de Mallorca, Spain; 10 Internal Medicine Service, Hospital Regional Universitario, Málaga, Spain; 11 Internal Medicine Service, Hospital Universitario Dr Peset, Valencia, Spain; 12 Internal Medicine Service, Hospital General Universitario, Alicante, Spain; 13 Infectious Diseases Unit, Hospital Clínico Universitario, Valladolid, Spain; 14 Internal Medicine Service, Hospital POVISA, Pontevedra, Spain; 15 Infectious Diseases Unit, Hospital Universitario Central de Asturias, Oviedo, Spain; Stanford University School of Medicine, UNITED STATES

## Abstract

**Background:**

Valproic acid (VPA) has shown beneficial effects in vitro against SARS-CoV-2 infection, but no study has analyzed its efficacy in the clinical setting.

**Methods:**

This multicenter, retrospective study included 165 adult patients receiving VPA at the time of admission to hospital, and 330 controls matched for sex, age and date of admission. A number of clinical, outcome and laboratory parameters were recorded to evaluate differences between the two groups. Four major clinical endpoints were considered: development of lung infiltrates, in-hospital respiratory worsening, ICU admissions and death.

**Results:**

VPA-treated patients had higher lymphocyte (P<0.0001) and monocyte (P = 0.0002) counts, and lower levels of diverse inflammatory parameters, including a composite biochemical severity score (P = 0.016). VPA patients had shorter duration of symptoms (P<0.0001), were more commonly asymptomatic (P = 0.016), and developed less commonly lung infiltrates (65.8%/88.2%, P<0.0001), respiratory worsening (20.6%/30.6%, P = 0.019) and ICU admissions (6.1%/13.0%, P = 0.018). There was no difference in survival (84.8%/88.8%, P = 0.2), although death was more commonly related to non-COVID-19 causes in the VPA group (36.0%/10.8%, P = 0.017). The cumulative hazard for developing adverse clinical endpoints was higher in controls than in the VPA group for infiltrates (P<0.0001), respiratory worsening (P<0.0001), and ICU admissions (P = 0.001), but not for death (0.6). Multivariate analysis revealed that VPA treatment was independently protective for the development of the first three clinical endpoints (P = 0.0002, P = 0.03, and P = 0.025, respectively), but not for death (P = 0.2).

**Conclusions:**

VPA-treated patients seem to develop less serious COVID-19 than control patients, according to diverse clinical endpoints and laboratory markers.

## Introduction

In December 2019 a cluster of severe pulmonary infections requiring hospital admission and, in many cases, mechanical ventilation in workers of the wet market of Wuhan, Hubei province, China, was reported [1]. These pulmonary infections were due to a new beta-coronavirus, named SARS-CoV-2, responsible for the so called COVID-19. Since then this infection became pandemic. As for November 7, 2021, 248 million of patients with confirmed COVID-19 had been reported worldwide, including 5 million of deaths [2]. The COVID-19 pandemic has collapsed the health services of most of the countries throughout the world, even of the wealthiest, and ruined the world economy. In spite of intensive efforts by the pharmaceutical companies to obtain an effective antiviral drug against COVID-19, most of the drugs tested so far proved ineffective or even harmful such as hydroxychloroquine, azithromycin or lopinavir/ritonavir [3–5]. Remdesivir, an antiviral previously designed to treat Ebola virus, is the only licensed drug with some antiviral effect on COVID-19. Remdesivir treatment seems to reduce the hospital admission days, but neither affects mortality nor decreases the viral load from nasopharyngeal or pulmonary exudates [6, 7]. So far, only dexamethasone has proved clinical benefit in reducing deaths in certain subgroups of patients [8] and fluvoxamine, a serotonin reuptake inhibitor, in reducing hospitalization among high-risk outpatients with early infection [9]. Molnupiravir might become the first oral antiviral COVID treatment in the coming months. This antiviral, a prodrug of the synthetic nucleoside derivative N4-hydroxycytidine (also called EIDD-1931), forces the SARS-CoV-2 to mutate itself to death by introducing copying errors during viral RNA replication [10].

Valproic acid (VPA) is a branched, short-chain fatty acid, and the 2-n-propyl derivative of valeric acid. VPA has been used for more than 60 years to treat epilepsy and other neurologic and psychiatric diseases including bipolar disorders, neuropathic pain and migraine. In the last years an antiviral effect of VPA on herpes viruses, including herpes simplex virus 1 and 2, varicella zoster virus, Epstein-Barr virus and cytomegalovirus, has been reported [11–16]. Very recently an antiviral effect of VPA on dengue and SARS-CoV-2 has also been suggested in a virtual simulation study [17]. Interestingly, VPA administration decreased the LPs-induced lung inflammation in murine models, suggesting a protective effect of VPA on acute lung injury [18, 19], and suppressed TNF-alpha and IL-6 production via inhibition of NF-kappaB activation, pointing towards a modulating role of VPA on immune responses [20].

VPA also inhibits histone deacetylase 2 (HDAC2), responsible for the deacetylation of lysine residues on the N-terminal region of the core histones, and HDAC2 inhibition modifies gene transcription. Thus VPA inhibits the expression of angiotensin-converting enzyme (ACE-2) receptors that are the entry door for SARS-CoV-2 into the cells [21]. Likewise, VPA decreases the expression of IL-6 in endothelial cells, a key element of the cytokines storm, the inflammatory event leading to lung infiltrates and pulmonary insufficiency in severe COVID-19, and also decreases the expression of the intercellular adhesion molecule 1(ICAM-1), a cell surface glycoprotein that enhances viral adhesion to the capillary endothelial cells, and facilitates intravascular thrombosis, a severe complication of COVID-19 [21].

A recent study using diverse cell lines found that VPA blocks three important processes involved in the severity of COVID-19 infection: the drug downregulates the expression of ACE2 and neuropili-1 (NRP1) decreasing viral infectivity, it reduces viral yields, probably because of alterations of the virus budding or virions stability, and it diminishes the inflammatory response to the viral infection [22].

Thus VPA is in an excellent position to be tested in patients with COVID-19 due to its antiviral and inflammatory properties. However, no study to date has evaluated the possible usefulness of VPA in the clinical setting.

Because many individuals are exposed for years to VPA to treat their neuropsychiatric disorders, the easiest way to test VPA efficacy on COVID-19 is to analyze the presentation, evolution and outcome of COVID-19 in patients who are taking VPA at the time of admission to the hospital. In this line of thought, we have carried out a retrospective, observational, multicenter study to compare the clinical and lab characteristics, course, treatments and outcomes of patients exposed and not exposed to VPA, matched by age, sex, and COVID-19 pandemic wave.

## Patients and methods

This retrospective, case control study was developed in the following 14 Spanish hospitals, totalizing 10,917. hospital beds and providing care for a population of 4,612,712 inhabitants, about one-tenth of the total population of our country: Hospital de la Santa Creu i Sant Pau (Barcelona), Hospital General Universitario Gregorio Marañón (Madrid), Hospital Universitario Fundación Jiménez Díaz (Madrid), Hospital Universitario Central de Asturias (Oviedo), Hospital Universitari Son Espases (Palma de Mallorca), Hospital Universitario 12 de Octubre (Madrid), Hospital Universitario La Princesa (Madrid), Hospital Universitario y Politécnico La Fe (Valencia), Hospital Son Llàtzer (Palma de Mallorca), Hospital Regional Universitario (Málaga), Hospital Clínico Universitario (Valladolid), Hospital General Universitario (Alicante), Hospital Universitario Dr Peset (Valencia), and Hospital POVISA (Vigo).

Patients were considered for inclusion if admitted to the participating hospitals during a period of one year, from 1 March 2020 to 28 February 2021, covering the three major epidemic waves suffered in Spain from 2020 to 2021 (spring, autumn and winter). Cases were identified through electronic records. The first step was the detection of all in-hospital VPA prescriptions by means of the Pharmacy departments of the respective hospitals. The patients thus identified were scrutinized for simultaneous SARS-CoV-2 infection by means of microbiology records, PCR, antigen and serology results, codified diagnoses and/or discharge reports.

Adult patients fulfilling the criteria of being treated with VPA at the time of arrival, SARS-CoV-2 infection and hospital admission were included in the study as cases. Two controls were selected for each case matched by age, sex and date of admission, as each of these three features might have influence on the outcome. Particular attention was devoted to the choice of the controls to prevent selection bias. Thus, control patients were selected at each participating hospital from electronic, non-clinical records, in which only the date of birth, gender and date of admission were available. Therefore, no clinical, laboratory or outcome data were known at the time of selection of the controls. The two closest patients to the index VPA case according to these three matching factors were selected as controls.

A number of demographic, comorbid, microbiological, clinical, diagnostic, laboratory, imaging, hospitalization, prognostic, and therapeutic data were collected from medical records and compared in cases and controls. As many patients lacked diverse biochemical determinations, a composite biochemical severity score was constructed to evaluate the impact of the infection from a biochemical point of view in those parameters commonly affected by COVID-19 (C-reactive protein, alanine aminotransferase, lactic dehydrogenase, ferritin, procalcitonin, interleukin-6 (IL-6), D-dimer, troponin and N-terminal pro B-type natriuretic peptide (NT-proBNP)). This biochemical score was calculated for each patient by dividing the available determinations into their respective upper normal ranges, adding the resulting values, and dividing this result into the number of parameters measured.

Patients were also classified into two categories according to their respiratory evolution during the in-hospital stay. Worsening respiratory status was considered if the patients required additional oxygen supplementation or mechanical ventilation in the clinical ward or Intensive Care Unit (ICU) relative to their requirements at the time of arrival at the Emergency Department, whereas no worsening was considered if the need for oxygen supplementation did not increase during hospitalization. Four major clinical endpoints were considered for analysis: development of lung infiltrates, in-hospital respiratory worsening, ICU admission, and death.

This was a retrospective, observational study using anonymized data from electronic records, based on patients who had undergone routine clinical care for COVID-19. Therefore, according to the Spanish law, no formal written informed consent was obtained from the patients, who had been already discharged from the hospital by then and some of whom even had died. The study was approved by the Research Ethics Committee of the Principality of Asturias, Spain, which also granted a formal waiver of requiring the consent from the patients.

### Statistical analysis

As the distribution of continuous variables was non-Gaussian, according to the Kolmogorov-Smirnov test, original values underwent natural logarithmic transformation for analysis. The reported values are the result of back-transformation into the original units, and are expressed as geometric mean and 95% CI. Variables not suitable for logarithmic transformation, such as those containing 0 or negative values, are reported as median, IQ range. The *t*-test and the Mann-Whitney U test were used for the comparison of continuous variables, according to the nature of the variable. Proportions were compared with the chi-square test or Fisher’s exact test, as appropriate. Correlations among doses and duration of VPA treatment with other parameters were assessed with the Pearson’s correlation coefficient. Receiver operating characteristic (ROC) curves and the area under the ROC curve (AUROC) were calculated to identify the laboratory parameters more discriminant of clinical endpoints, whereas the differential association between cases and controls with these endpoints was analyzed with the Kaplan-Meier hazard function, using the log-rank Mantel-Cox test to assess their statistical significance. Logistic regression analysis models were constructed to identify the factors independently associated with clinical outcomes. SPSS v. 25 software (IBM Corp., Armonk, NY, USA) was used for statistical calculations. A P value <0.05 for a two-tailed test was considered statistically significant.

## Results

A total of 165 cases and 330 controls were included in the study. The mean age was 60.1 years (95% CI 58.3–61.9) and the male to female ratio was 1.3/1. Regarding the epidemic waves, 159 patients (32.1%) were admitted during the first, 213 (43.0%) during the second and 123 (24.9%) during the third wave. We also calculated the incidences of hospital admissions by dividing the corresponding cases (165 for VPA patients and 43,637 for overall COVID-19 patients) into the total population covered by the 14 participating hospitals (4,612,712). Thus, the estimated incidence of hospital admissions because of COVID-19 of the VPA-treated patients during the year of study was 3.58/100,000 individuals, whereas the overall incidence of COVID-19 cases admitted to the participant hospitals during the same period was 946/100,000 individuals. Therefore, admissions of VPA-treated patients represented 0.38% of the total admissions for COVID-19.

Table 1 shows the demographic features, habits, comorbidities and treatments at the hospital arrival. There were no significant differences between the two groups in any of the parameters evaluated, with the only exception of a somewhat higher frequency of antihypertensive treatment with angiotensin receptor blockers in the control group, which was only marginally significant. However, this treatment was not significantly associated with the development of lung infiltrates (P = 0.3), respiratory worsening (P = 0.2), ICU admission (P = 0.2) or death (P = 0.9).

**Table 1:** Demography, habits, comorbidities and treatment at the time of hospital arrival.

		All	VPA cases	Controls	P value
		(n = 495)	(n = 165)	(n = 330)	
**Demography & anthropometry**					
Gender	Male	282 (57.0%)	94 (57.0%)	188 (57.0%)	1
	Female	213 (43.0%)	71 (43.0%)	142 (43.0%)	
Age	years (n = 495)	60.1 (58.3–61.9)	59.9 (56.5–62.8)	60.3 (58.2–62.6)	0.8
Weight	kg (n = 279)	77.3 (75.4–79.2	77.2 (73.6–80.9)	77.3 (75.1–79.6)	0.9
Body mass index	(n = 232)	28.34 (27.62–29.07)	27.96 (26.59–29.41)	28.52 (27.70–29.37)	0.5
**Comorbidities / habits**					
Diabetes	Yes	95 (19.2%)	30 (18.2%)	65 (19.7.0%)	0.7
	No	400 (80.8%)	135 (81.8%)	265 (80.3%)	
Hypertension	Yes	231 (46.7%)	68 (41.2%)	163 (49.4%)	0.09
	No	264 (53.3%)	97 (58.8%)	167 (50.6%)	
Immunosuppression	Yes	49 (9.9%)	19 (11.5%)	30 (9.1%)	0.4
	No	446 (90.1%)	146 (88.5%)	300 (90.9%)	
Immunosuppression, causes*	Cancer	28 (57.1%)	8 (42.1%)	20 (66.7%)	0.15
	HIV	6 (12.2%)	4 (21.1%)	2 (6.7%)	
	Immunosuppressors	9 (18.4%	3 (15.8%)	6 (20.0%)	
	Other conditions	6 (12.2%)	4 (21.1%)	2 (6.7%)	
Smoking	Yes	32 (6.5%)	15 (9.1%)	17 (5.2%)	0.09
	No	461 (93.5%)	149 (90.0%)	310 (94.8%)	
Alcohol (>50 gr/d for ≥5 years)	Yes	20 (4.1%)	7 (4.3%)	13 (4.0%)	0.9
	No	472 (95.9%)	156 (95.7%)	316 (96.0%)	
Antiepileptic/psychoactive therapy*					
VPA doses	mg/day (n = 165)	-	1098 (1001–1196)	-	-
VPA therapy duration	years (n = 142)	-	6.77 (5.75–7.79)	-	-
Reason for VPA therapy	Epilepsy	-	84 (50.9%)	-	-
	Mental disorder	-	75 (45.5%)	-	
	Other		6 (3.6%)		
Concomitant use of other psychoactive drugs	Yes	-	68 (41.2%)	-	-
	No	-	97 (58.8%)	-	
Antihypertensive therapy*					
Angiotensin-converting enzyme inhibitors	Yes	75 (33.8%)	16 (23.9%)	59 (38.1%)	0.04
	No	147 (66.2%)	51 (76.1%)	96 (61.9%)	
Angiotensin receptor blockers	Yes	84 (37.8%)	21 (31.3%)	63 (40.6%)	0.19
	No	138 (62.2%)	46 (68.7%)	92 (59.4%)	
Other antihypertensive drugs	Yes	140 (63.1%)	43 (64.2%)	97 (62.6%)	0.8
	No	82 (36.9%)	24 (35.8%)	58 (37.4%)	

VPA denotes Valproic acid.

Values are expressed as mean (95% CI) or %.

* Only in patients who fulfilled the condition.

Table 2 depicts the clinical features, radiological findings, laboratory determinations at admission and diagnostic procedures for SARS-CoV-2 infection. As compared to VPA cases, controls were more likely to be symptomatic and to have pulmonary infiltrates. Controls had also longer duration of symptoms and time to infiltrates detection and higher values of CRP, transaminases, ferritin and the biochemical severity score than the VPA group. Regarding hematological parameters, controls had significantly lower lymphocyte, monocyte and basophil counts than VPA-treated patients.

**Table 2:** Clinical, radiologic, laboratory and diagnostic procedures at admission.

		All	VPA cases	Controls	P value
		(n = 495)	(n = 165)	(n = 330)	
**Clinical features**					
Duration of symptoms*	days (n = 472)	5.0 (2.0–9.0)	3.0 (1.0–7.0)	7.0 (3.0–10.0)	<0.0001
Asymptomatic	Yes	23 (4.6%)	13 (7.9%)	10 (3.0%)	0.016
	No	472 (95.4%)	152 (92.1%)	320 (97.0%)	
Temperature	°C (n = 488)	37.03 (36.90–37.16)	37.03 (36.88–37.19)	37.03 (36.84–37.21)	1
Respiratory rate	per minute (n = 391)	19.80 (19.308–20.31)	20.24 (19.32–21.22)	19.59 (18.99–20.31)	0.2
Heart rate	per minute (n = 486)	87.65 (86.11–89.22)	86.74 (83.99–89.57)	88.10 (86.24–90.00)	0.4
Oxygen saturation	% (n = 491)	93.5 (93.0–94.0)	93.2 (92.4–94.1)	93.6 (93.1–94.2)	0.4
pO_2_	mm Hg (n = 243)	70.2 (67.4–73.1)	69.2 (64.1–74.8)	70.6 (67.4–74.1)	0.6
Need for supplementary oxygen	Yes	282 (57.0%)	106 (64.2%)	176 (53.3%)	0.02
	No	213 (43.0%)	59 (35.8%)	154 (46.7%)	
Pulmonary infiltrates	Yes	390 (80.7%)	106 (65.8%)	284 (88.2%)	<0.0001
	No	93 (19.3%)	55 (34.2%)	38 (11.8%)	
Bilateral pulmonary infiltrates*	Yes	328 (84.1%)	87 (82.1%)	241 (84.9%)	0.5
	No	62 (15.9%)	19 (17.9%)	43 (15.1%)	
Time to infiltrates detection since the onset of symptoms* ^§^	days (n = 384)	6.0 (3.0–9.0)	5.0 (2.0–8.0)	7.0 (4.0–10.0)	0.002
**Laboratory blood determinations**					
Total leukocyte count	cells/μL (n = 485)	6411.3 (6163.1–6669.6)	6439.2 (5995.1–6916.2)	6397.6 (6100.7–6700.9)	0.9
Neutrophils	cells/μL (n = 485)	4521.4 (4303.0–4750.8)	4249.4 (3888.9–4643.3)	4662.9 (4393.0–4949.4)	0.08
Lymphocytes	cells/μL (n = 485)	1033.3 (985.2–1083.7)	1193.7 (1089.6–1307.7)	961.8 (911.4–1014.8)	<0.0001
Monocytes	cells/μL (n = 485)	459.8 (434.4–486.8)	535.8 (483.9–593.1)	426.2 (398.5–455.9)	0.0002
Eosinophils	cells/μL (n = 485)	7.73 (6.58–9.09)	9.73 (7.19–13.18)	6.90 (5.70–8.34)	0.049
Basophils	cells/μL (n = 485)	8.89 (7.83–10.08)	13.42 (10.79–16.68)	7.24 (6.23–8.41)	<0.0001
C-reactive protein	mg/L (n = 474)	46.26 (41.28–51.85)	35.93 (29.09–44.39)	52.56 (46.02–60.02)	0.002
Procalcitonin	ng/mL (n = 255)	0.10 (0.09–0.11)	0.10 (0.08–0.13)	0.10 (0.09–0.12)	0.9
Aspartate aminotransferase	U/L (n = 357)	32.66 (30.49–34.99)	29.43 (26.07–33.21)	34.39 (31.64–37.38)	0.036
Alanine aminotransferase	U/L (n = 453)	26.21 (24.49–28.06)	21.63 (19.09–24.51)	28.82 (26.63–31.20)	0.0001
Creatine kinase	U/L (n = 298)	102.6 (91.4–115.1)	115.0 (90.9–145.5)	96.5 (85.2–109.3)	0.15
Lactate dehydrogenase	U/L (n = 398)	293.6 (282.6–304.6)	280.4 (261.0–301.2)	299.4 (286.6–312.8)	0.11
Ferritin	ng/mL (n = 295)	460.4 (409.2–518.0)	352.5 (286.8–433.1)	524.7 (455.6–604.4)	0.002
IL-6	pg/mL (n = 210)	18.75 (15.16–23.18)	18.85 (12.72–27.94)	18.70 (14.50–24.11)	0.9
D-dimer	ng/mL (n = 392)	585.3 (524.5–648.8)	593.4 (489.2–719.8)	578.6 (508.99–657.7)	0.8
Troponin	ng/L (n = 190)	9.27 (8.05–10.67)	9.60 (7.35–12.53)	9.13 (7.72–10.79)	0.7
NT-proBNP	pg/mL (n = 132)	262.0 (199.3–344.3	278.5 (179.6–431.9)	254.1 (178.7–361.3)	0.8
Biochemical severity score	(n = 484)	3.19 (2.94–3.45)	2.77 (2.37–3.24)	3.42 (3.12–3.74)	0.016
**Diagnostic procedures for SARS-CoV-2 infection**					
Nasopharyngeal PCR	Yes	451 (94.0%)	154 (93.9%)	297 (94.0%)	1
	No	29 (6.0%)	10 (6.1%)	19 (6.0%)	
Nasopharyngeal viral load*	log copies/1000 cells (n = 45)	6.437 (6.010–6.864)	6.541 (5.877–7.204)	6.380 (5.798–6.961)	0.7
Other samples PCR	Yes	4 (2.2%)	0 (0.0%)	4 (3.4%)	0.14
	No	174 (97.8%)	61 (100.0%)	113 (96.6%)	
Nasopharyngeal antigen	Yes	67 (63.8%)	19 (59.4%)	48 (65.8%)	0.5
	No	38 (36.2%)	13 (40.6%)	25 (34.2%)	
Positive IgM serology	Yes	37 (39.4%)	12 (30.8%)	25 (45.5%)	0.15
	No	57 (60.6%)	27 (69.2%)	30 (54.5%)	
Positive IgG serology	Yes	45 (37.5%)	17 (34.7%)	28 (39.4%)	0.6
	No	75 (62.5%)	32 (65.3%)	43 (60.6%)	
Positive IgM and/or IgG	Yes	51 (42.9%)	18 (36.7%)	33 (47.1%)	0.3
	No	68 (57.1%)	31 (63.3%)	37 (52.9%)	

VPA denotes Valproic acid.

Values are expressed as mean (95% CI), ^§^median (IQ range) or %.

* Only in patients who fulfilled the condition.

The severity biochemical score was also significantly increased in patients with lung infiltrates (mean 3.63, 95% CI 3.35–3.94, vs 1.92 95% CI 1.56–2.36, P<0.0001), respiratory worsening (mean 3.87, 95% CI 3.37–4.46 vs 2.96, 95% CI 2.68–3.25, P = 0.003), ICU admission (mean 5.49, 95% CI 4.66–6.47 vs 2.98, 95% CI 2.73–3.25, P<0.0001) and in those who died (mean 5.82, 95% CI 4.69–7.24 vs 2.93, 95% CI 2.69–3.18, P<0.0001). The AUROCs for these four outcomes were 0.687 (95% CI 0.622–0.752) P<0.0001; 0.588 (0.533–0.642) P = 0.003; 0.700 (0.638–0.761) P<0.0001; and 0.719 (0.646–0.793) P<0.0001, respectively. In multivariate analyses this biochemical severity score was independently predictive of the development of infiltrates (OR 1.641, 95% CI 1.161–2.319, P = 0.005), ICU admission (OR 1.939, 1.030–3.649, P = 0.04) and death (OR 2.361, 1.331–4.190, P = 0.003), but not of respiratory worsening (P = 0.2).

VPA daily dosage only correlated with higher lymphocyte (n = 161, r = 0.20, P = 0.01) and mononuclear cell counts (n = 161, r = 0.19, P = 0.017), whereas duration of VPA treatment correlated with higher procalcitonin levels (n = 69, r = 0.30, P = 0.01), lower nasopharyngeal viral load (n = 7, r = -0.80, P = 0.029), and lower aspartate aminotransferase levels (n = 96, r = -0.24, P = 0.02). Dosage of VPA was not significantly associated with the development of lung infiltrates (P = 0.3), respiratory worsening (P = 0.9), ICU admission (P = 0.2) or death (P = 0.6).

Table 3 shows the management and treatments during the hospital stay. There were no significant differences in the management of the two groups, with the exception of a significantly higher rate of dexamethasone treatment, as well as a trend towards a higher rate of tocilizumab therapy, in controls as compared to VPA patients.

**Table 3:** In-hospital management and treatment.

		All	VPA cases	Controls	P value
		(n = 495)	(n = 165)	(n = 330)	
Supplementary oxygen in clinical ward/ICU	Yes	352 (71.1%)	126 (76.4%)	226 (68.5%)	0.07
	No	143 (28.9%)	39 (23.6%)	104 (31.5%)	
**Treatment**					
Dexamethasone	Yes	192 (38.9%)	53 (32.3%)	139 (42.1%)	0.03
	No	302 (61.1%)	111 (67.7%)	191 (57.9%)	
Dexamethasone duration*	days (n = 192)	7.45 (6.78–8.18)	7.06 (5.70–8.75)	7.60 (6.85–8.42)	0.5
Methylprednisolone	Yes	141 (28.5%)	40 (24.2%)	101 (30.7%)	0.13
	No	353 (71.5%)	125 (75.8%)	228 (69.3%)	
Methylprednisolone duration*	days (n = 139)	3.92 (3.44–4.47)	4.67 (3.59–6.08)	3.66 (3.15–4.26)	0.1
Tocilizumab	Yes	47 (9.5%)	10 (6.1%)	37 (11.2%)	0.07
	No	448 (90.5%)	155 (93.9%)	293 (88.8%)	
Tocilizumab duration*	days (n = 46)	1.16 (1.04–1.30)	1.41 (0.87–2.29)	1.10 (1.01–1.20)	0.07
Remdesivir	Yes	29 (5.9%)	8 (4.8%)	21 (6.4%)	0.5
	No	466 (94.1%)	157 (95.2%)	309 (93.6%)	
Remdesivir duration*	days (n = 28)	5.04 (4.56–5.58	4.46 (3.40–5.84)	5.30 (4.77–5.88)	0.12
Low-molecular weight heparin (≥0,5 mg/Kg/d)	Yes	420 (84.8%)	140 (84.8%)	280 (84.8%)	1
	No	75 (15.2%)	25 (15.2%)	50 (15.2%)	
Heparin duration*	days (n = 418)	9.52 (8.81–10.29)	9.80 (8.51–11.28)	9.39 (8.55–10.30)	0.6
**Other commonly used drugs**					
Hydroxychloroquine	Yes	112 (22.6%)	39 (23.6%)	73 (22.1%)	0.7
	No	383 (77.4%)	126 (76.4%)	257 (77.9%)	
Azithromycin	Yes	103 (20.8%)	29 (17.6%)	74 (22.4%)	0.2
	No	392 (79.2%)	136 (82.4%)	256 (77.6%)	
Lopinavir/ritonavir	Yes	53 (10.7%)	13 (7.9%)	40 (12.1%)	0.15
	No	442 (89.3%)	152 (92.1%)	290 (87.9%)	
Ceftriaxone	Yes	100 (20.2%)	38 (23.0%)	62 (18.8%)	0.3
	No	395 (79.8%)	127 (77.0%)	268 (81.2%)	

VPA denotes Valproic acid.

Values are expressed as median (IQ range) or %.

* Only in patients who fulfilled the condition.

Table 4 describes the in-hospital course and outcomes. Controls experienced significantly higher rates of ICU admissions and respiratory worsening during the hospital stay than VPA-treated patients. On the contrary, no differences in the overall death rate were observed between cases and controls, although deaths specifically related to COVID-19 causes were significantly less common in the VPA than in the control groups in those patients who died.

**Table 4:** In-hospital course and outcomes.

		All	VPA cases	Controls	P value
		(n = 495)	(n = 165)	(n = 330)	
Nosocomial acquisition	Yes	8 (1.6%)	6 (3.6%)	2 (0.6%)	0.01
	No	487 (98.4%)	159 (96.4%)	328 (99.4%)	
Duration of in-hospital stay	days (n = 495)	9.07 (8.41–9.77)	9.76 (8.49–11.21)	8.74 (8.00–9.55)	0.17
Time to discharge since the onset of symptoms* ^§^	days (n = 472)	16.0 (11.0–22.0)	15.0 (10.0–21.75)	16.0 (12.0–22.0)	0.07
Time to negative PCR since the onset of symptoms* ^§^	days (n = 159)	20.0 (14.0–32.0)	18.0 (13–29.5)	21.0 (14.0–33.0)	0.3
Respiratory worsening	Yes	135 (27.3%)	34 (20.6%)	101 (30.6%)	0.019
	No	360 (72.7%)	131 (79.4%)	229 (69.4%)	
Intensive Care Unit admission	Yes	53 (10.7%)	10 (6.1%)	43 (13.0%)	0.018
	No	442 (89.3%)	155 (93.9%)	287 (87.0%)	
Duration of ICU stay*	days (n = 53)	14.06 (10.94–18.07)	17.45 (9.21–33.04)	13.35 (10.06–17.72)	0.4
Need for mechanical ventilation	Yes	58 (11.7%)	14 (8.5%)	44 (13.3%)	0.11
	No	437 (88.3%)	151 (91.5%)	286 (86.7%)	
Complications	Yes	122 (24.6%)	43 (26.1%)	79 (23.9%)	0.6
	No	373 (75.4%)	122 (73.9%)	251 (76.1%)	
Sequelae at discharge	Yes	39 (8.9%)	16 (11.4%)	23 (7.7%)	0.2
	No	399 (91.1%)	124 (88.6%)	275 (92.3%)	
Positive IgM serology at discharge	Yes	66 (57.9%)	19 (47.5%)	47 (63.5%)	0.098
	No	48 (42.1%)	21 (52.5%)	27 (36.5%)	
Positive IgG serology at discharge	Yes	129 (75.4%)	46 (74.2%)	83 (76.1%)	0.8
	No	42 (24.6%)	16 (25.8%)	26 (23.9%)	
Positive IgM and/or IgG at discharge	Yes	129 (75.9%)	47 (75.8%)	82 (75.9%)	1
	No	41 (24.1%)	15 (24.2%)	26 (24.1%)	
Outcome	Survival	433 (87.5%)	140 (84.8%)	293 (88.8%)	0.2
	Death	62 (12.5%)	25 (15.2%)	37 (11.2%)	
Death caused by COVID-19*	Yes	49 (79.0%)	16 (64.0%)	33 (89.2%)	0.017
	No	13 (21.0%)	9 (36.0%)	4 (10.8%)	

VPA denotes Valproic acid.

Values are expressed as mean (95% CI), ^§^median (IQ range) or %.

* Only in patients who fulfilled the condition.

These favorable effects on certain clinical endpoints were not due to the higher rate of asymptomatic patients at admission in the VPA treated group, because significant differences were also observed when the asymptomatic patients were excluded from the analysis for lung infiltrates (P<0.0001), respiratory worsening (P = 0.026), and ICU admissions (P = 0.015).

ROC curves revealed that lymphocyte and monocyte counts had similar predictive values and were the most discriminant hematological cell types for the four clinical endpoints, as well as for VPA treatment. The combination of these two mononuclear cells was moderately but significantly discriminatory for the development of lung infiltrates (AUROC 0.661, 95% CI 0.598–0.725, P<0.0001), respiratory worsening (AUROC 0.584, 0.528–0.640, P = 0.004), ICU admissions (AUROC 0.677, 0.602–0.753, P<0.0001), and death (AUROC 0.604, 0.522–0.687, P = 0.009), as well as to identify the VPA group (AUROC 0.642, 0.589–0.696, P<0.0001).

**Fig 1:**
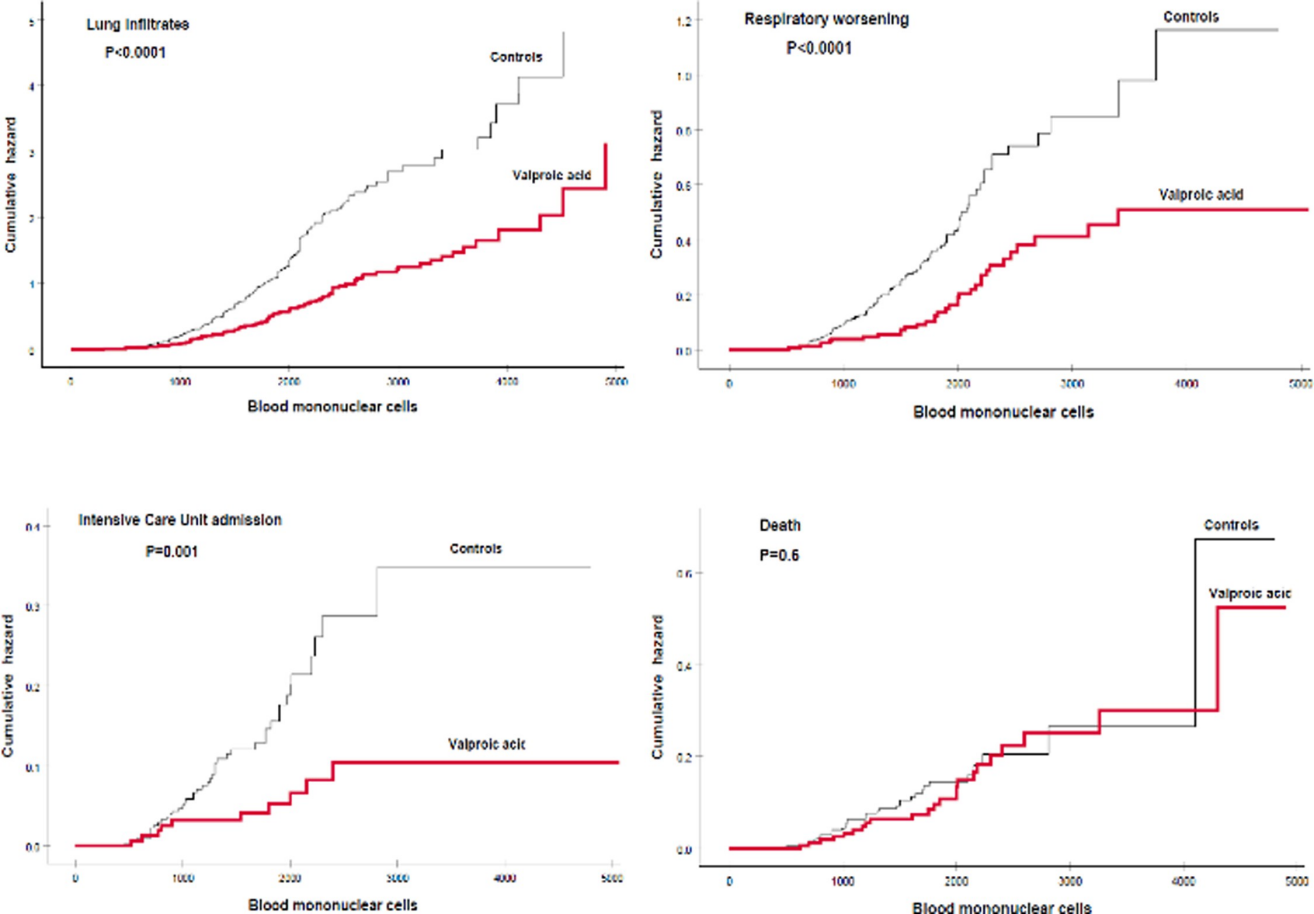
Hazard functions for the development of diverse clinical outcomes according to the blood mononuclear cell count (cells/μ).

Taking into account the somewhat better predictive value of the combination of these two mononuclear cell types, hazard functions were calculated for the development of the four clinical outcomes evaluated in case and control patients (Fig 1).

As seen in the Figure, control patients had significantly higher cumulative hazards for these adverse outcomes than VPA-treated individuals across the mononuclear cell range for the development of lung infiltrates, respiratory worsening and ICU admissions, but not for death. Regarding biochemical parameters, control patients also had higher cumulative hazards than VPA cases according to the biochemical severity index, although the association was somewhat less marked: lung infiltrates, P = 0.03; respiratory worsening, P = 0.06; ICU admissions, P = 0.027; death, P = 0.4.

To identify the factors independently predictive of each of the four clinical endpoints evaluated, backward stepwise logistic regression analyses were carried out, including as explanatory variables those parameters that were significantly associated with the respective clinical endpoint in the univariate analyses, although VPA treatment was entered into the analysis for death despite the lack of univariate association. In these models, which adequately fitted the data according to the Hosmer–Lemeshow goodness-of-fit statistic, VPA treatment was independently protective for the development of lung infiltrates (OR 0.309, 95% CI 0.168–0.568, P = 0.0002), in-hospital respiratory worsening (OR 0.508, 95% CI 0.273–0.945, P = 0.03), and ICU admissions (OR 0.251, 95% CI 0.075–0.843, P = 0.025), whereas it was not predictive of death (P = 0.2).

## Discussion

To our knowledge, this is the first clinical study that evaluates the effect of VPA therapy on patients with COVID-19. We found that VPA-treated patients, as compared to control patients matched for age, sex and admission date, developed less commonly and had lower hazards for the development of pulmonary infiltrates, in-hospital respiratory worsening and ICU admissions, as well as had higher lymphocyte and monocyte counts and lower levels of diverse biochemical inflammatory markers. Beneficial effects that were confirmed by multivariate analyses after adjusting for covariates.

Rates of ICU admissions should be interpreted cautiously, as diverse factors such as medical judgment, hospital policies and bed availability, among others, may influence this decision. However, our findings of a substantial reduction in ICU admissions in the VPA group seem consistent. Firstly, because these confounding factors would expectedly affect to both groups. Secondly, because of the degree of improvement, as control patients more than doubled the rate of ICU admission as compared to the VPA group. Finally, improvements in other clinical endpoints, such as development of lung infiltrates and respiratory worsening, as well as substantial reductions in diverse hematological and biochemical parameters known to be associated with severe COVID-19 were also objective indicators or a more benign disease in the VPA-treated patients.

Despite these improvements in clinical endpoints and laboratory parameters, no improvement was observed in survival. However, it should be considered that all VPA-treated patients had additional neuropsychiatric comorbidities, some of them serious, which may account for additional deaths, both related and unrelated to COVID-19. In fact, we found that deaths not strictly related to-COVID-19 were significantly more common in the VPA than in the control group. In addition, control patients received more commonly than VPA patients some treatments aimed to improve COVID-19 outcomes, such as tocilizumab, methylprednisolone and, particularly, dexamethasone, a drug that has proved to be efficacious in reducing mortality rates [8], and that was administered significantly more frequently to control patients.

Epilepsy was the most common cause of VPA treatment in our study, involving 51% of the patients. If patients with epilepsy suffer from more severe COVID-19 is controversial, as exemplified by two large studies, one of which found that the infection was more prevalent, required more hospital and ICU admissions and was more lethal in epileptic patients than in the general population [23], whereas the other did not find differences between these two groups [24].

Low lymphocyte and monocyte counts have been associated with serious COVID-19 [25–30]. In this regard, we found significantly higher lymphocyte and monocyte and lower neutrophil counts in VPA-treated than in control patients, and there was also a positive correlation between VPA doses and lymphocyte counts, supporting the beneficial effects of such a therapy. A non-COVID-19 study also found higher lymphocyte and lower neutrophil counts in patients treated with VPA than in those treated with other antiepileptic drugs, although in this study there was a similar negative correlation between VPA plasma levels and both neutrophil and lymphocyte counts [31]. The preservation of the blood mononuclear cells in VPA treated patients may have contributed to the clinical benefits observed in our study. Benefits of VPA treatment that are also supported by experimental studies [18, 19, 22] and by our findings of lower levels of some inflammatory biochemical parameters, including the biochemical severity score, as well as the lower hazards for developing certain adverse clinical endpoints according to the blood mononuclear counts. Therefore, the immunomodulatory and anti-inflammatory effects of VPA were presumably responsible for the improvements in these clinical endpoints.

From our study we cannot derive which would be the optimal VPA dosage for obtaining the best results. Although there was a significant positive correlation between daily dosage and lymphocyte counts, indicating that higher doses were associated with higher improvements in lymphocyte counts, such association was only moderate, there is little correlation between the dose administered and serum levels due to its high plasma protein binding [32], and no significant relationship was found between dosage and clinical endpoints, suggesting that low VPA doses may be similarly efficacious as larger ones.

Likewise, our study was based on VPA pre-treated patients and, therefore, the inferences about the effect that new treatments (e.g. at or before the onset of symptoms) could have on the course of the infection should be cautious. However, VPA is fully and rapidly absorbed, reaching peak plasma concentrations 1–4 hours after oral administration, and the steady state is reached 2–4 days after the onset of oral treatment, minutes in the case of intravenous administration [32, 33]. Therefore, its effects would expectedly be rapid, as also indicated by in vitro studies [18–22], and, consequently, its early administration might not only have a beneficial effect in hospitalized patients, but also a potential role in the prevention of hospital admissions.

Dexamethasone is nowadays the most effective therapy for serious COVID-19 due to its well-known anti-inflammatory properties. Treatment with this drug resulted in lower 28-day mortality in patients receiving either invasive mechanical ventilation or oxygen alone, but not in those who did not receive respiratory support [8]. On the other hand, the effect of the anti-IL-6 monoclonal antibody tocilizumab on patients hospitalized with COVID-19 is more controversial, although tocilizumab use may be associated with a short-term mortality and a reduction in the need for mechanical ventilation [34, 35]. Interestingly VPA-treated patients needed less dexamethasone and tocilizumab therapy, as well as methylprednisolone and remdesivir, compared to controls in our study, and had lower rates, not only of pulmonary infiltrates or respiratory worsening, but also ICU admission, an aspect that none of these drugs could demonstrate.

The main limitations of this study are those related to retrospective studies, including the lack of certain laboratory data in many patients and the inferences regarding causality, as well as the limited number of VPA-treated patients and the small number of deaths, which precluded us to perform a more precise mortality analysis. Also, we cannot entirely dismiss the existence of any occult VPA-unrelated factor that could favor the outcomes of the VPA-treated group, although this possibility is very unlikely and, in fact, these patients had additional comorbidities such as epilepsy, dementia and mental disorders. The main strengths of the study are its novelty and the enrollment of hospitalized patients throughout our country, covering about one-tenth of the Spanish population and the three COVID-19 major waves that ours and other Western European countries have suffered so far.

## Conclusions

We conclude that exposure to VPA seems to protect against the development of severe COVID-19, reducing the development of lung infiltrates, respiratory worsening and ICU admissions. This treatment was also associated with lower serum levels of diverse biochemical inflammatory markers, and higher peripheral blood lymphocyte and monocyte counts, objective laboratory parameters that support the clinical observations. However, large, prospective studies are needed to further clarify the role of VPA on COVID-19.
